# A new stonefly species (Plecoptera, Perlidae) from the Interior Highlands USA, with morphological and molecular comparison to other congeneric species

**DOI:** 10.3897/zookeys.858.33818

**Published:** 2019-07-01

**Authors:** Eric J. South, R. Edward DeWalt, Mark A. Davis, Michael Jared Thomas

**Affiliations:** 1 University of Illinois at Urbana-Champaign, Department of Entomology, 320 Morrill Hall, 505 S. Goodwin Ave., Urbana, IL 61801, USA University of Illinois at Urbana-Champaign Urbana United States of America; 2 University of Illinois, Prairie Research Institute, Illinois Natural History Survey, 1816 S. Oak St., Champaign, IL 61820, USA University of Illinois Champaign United States of America

**Keywords:** Arkansas, Nearctic, new species, Oklahoma, *Perlestasublobata* South & DeWalt, stonefly

## Abstract

Thirty-one species of Nearctic *Perlesta* Banks, 1906 (Plecoptera: Perlidae) are recognized. A new species is described from western Arkansas and eastern Oklahoma, USA, *Perlestasublobata* South & DeWalt, **sp. nov.**, from the adult male, adult female, and egg. *Perlestasublobata* males are differentiated from other congeners by a combination of a prominent ventral caecum and a distinct dorsal extension of the lateral sclerites of the aedeagus. A preliminary molecular phylogenetic hypothesis is proposed for *Perlesta* based on 17 congeners and three outgroup taxa using partial mitochondrial cytochrome c oxidase subunit I sequence data. Illustrations, stereomicroscope images, and scanning electron micrographs support the description and comparison to other *Perlesta*.

## Introduction

Needham and Claassen (1925) described *Perlesta* Banks, 1906 as a Nearctic genus of small, brown, triocellate stoneflies with yellow costal wing margins, long cerci, and highly variable coloration of the head and wing membrane. For over a century, the name of the type species of the genus, *Perlestaplacida* (Hagen, 1861), has been used for innumerable specimens that once critically reviewed, were revealed to encompass many cryptic species (Stark 1989, DeWalt et al. 2001). Stark (1989) revised the genus, removing several species from synonymy, describing seven new species, recognizing a total of 12 species, and providing the first useful key to the *P.placida* complex. Stark’s revision prompted additional work, with eight new species described over the next 14 years (Poulton and Stewart 1991, Kirchner and Kondratieff 1997, Stark and Rhodes 1997, DeWalt et al. 1998, Kondratieff and Baumann 1999, Kondratieff and Kirchner 2002, 2003). Subsequently, a revised taxonomic key was necessary, in which was included 21 Nearctic species (Stark 2004). The genus has since expanded to 31 Nearctic species through the works of Kondratieff et al. (2006, 2008, 2011), Kondratieff and Myers (2011), and Grubbs and DeWalt (2011, 2012, 2018).

Two species are recognized from China (Murányi and Li 2016) and undetermined nymphs have been reported from Costa Rica (Gutiérrez-Fonseca and Springer 2011). Described species could easily surpass 40, given the amount of presumed new, undescribed *Perlesta* material currently present in North American collections (Grubbs and DeWalt 2018).

At least eight species of *Perlesta* co-occur in the United States Interior Highlands, a mountainous region defined by the United States Geological Survey as encompassing southern Missouri, western Arkansas, eastern Oklahoma, and extreme southeastern Kansas (Omernik 1987). This area of the central United States was extensively examined for stoneflies by Poulton and Stewart (1991). Surprisingly, a remarkably distinct and undescribed *Perlesta* species from the Interior Highlands was revealed through recent examination of undetermined Arkansas material donated to the Illinois Natural History Survey (INHS) Insect Collection by the late Kenneth W. Stewart (DeWalt et al. 2018) and from eastern Oklahoma material borrowed from the K. C. Emerson Entomological Museum, Oklahoma State University (**OKSU**) at Stillwater. Using freshly collected and properly prepared specimens, we describe this new species, *Perlestasublobata* sp. nov., and compare it to similar regional congeners. Moreover, we provide the first comparative molecular study of the genus by exploring partial mitochondrial cytochrome c oxidase subunit I (COI) DNA sequence data to examine monophyly of the new species, delimit congeners, and construct a preliminary phylogeny.

The holotype male and all paratypes are deposited in the Illinois Natural History Survey (**INHS**) Insect Collection. Other material is deposited in the INHS Insect Collection with the exception of nine vials borrowed and returned to the OKSU Insect Collection.

## Materials and methods

### Collection and morphological analyses

**Figure 1.**
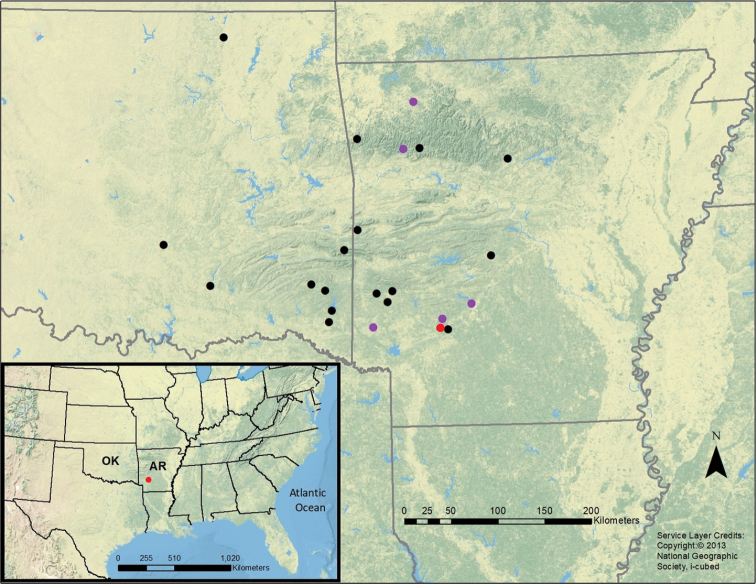
Collection sites (1932–2016) for *Perlestasublobata* sp. nov. in Oklahoma (OK) and Arkansas (AR), USA. Red circle represents type locality, 2016. Purple circles represent paratype localities, 2016.

Terminology of all stages follows Stark (1989). Fresh specimens of the new species (108 males and 40 females) were collected from six Arkansas stream systems, 13–19 June 2016 (Fig. 1). Methods included sweep netting during the day and ultraviolet light trapping at night. Live male specimens were anesthetized in a dry ice CO_2_ chamber and subsequently squeezed with forceps to evert the aedeagus, the source of the most informative morphological characteristics distinguishing species. All specimens were preserved in 95% EtOH. Select individuals of the fresh material and several related species were stack photographed and processed with a Zeiss AxioCam HRc Rev. 3 digital camera and Helicon Focus 6 software in the Sam W. Heads laboratory, INHS. The aedeagus and paraproct were sketched from the stereomicroscope images using Adobe Illustrator CC 2018. Scanning electron micrographs (SEM) of eggs and female terminalia were prepared at the Beckman Institute Microscopy Suite, University of Illinois by critical point drying, placing on an aluminum carbon disk, sputter coating with gold-palladium alloy, and imaged with a Thermo-Fisher FEI Quanta FEG 450 ESEM.

### Molecular studies

Genomic DNA from 20 *P.sublobata* specimens, 17 congeners, and three outgroup taxa was extracted using the Qiagen DNeasy Kit, amplified for a fragment of the mitochondrial gene encoding for the COI subunit via polymerase chain reaction using either primers LCO1490 and HCO2198 (Folmer et al. 1994) or jgLCO1490 and jgHCO2198 (Geller et al. 2013), and sequenced with Sanger technology at the University of Illinois W. M. Keck Core Sequencing Facility. Thermocycling conditions consisted of one 94 °C for 5 min denaturation cycle, 40 cycles at 94 °C for 45 s, 50 or 53 °C for 1 min, 72 °C for 1.5 min, and one 72 °C for 5 min extension cycle. Amplification success was verified with gel electrophoresis. Forward and reverse sequences were aligned to create contigs, and all 63 aligned contigs were truncated to a uniform length of 606 nucleotides, visually edited with Sequencher 5.4, aligned in MUSCLE 3.8, and the sequences and supporting data deposited in GenBank (Table 1). Sequences were tested to determine the model of evolution in jModelTest2 (Darriba et al. 2012), and a gamma distribution with a proportion of invariable sites was used to model rate variation across sites (invgamma). Akaike Information Criterion (AIC) results indicated that the General Time Reversible nucleotide substitution model (GTR+I+G) was best for the Maximum Likelihood and Bayesian analyses. These models were applied in subsequent phylogenetic tree generation analyses.

**Table 1.** **Haplotypes.** Description of COI haplotypes for 18 *Perlesta* species and three outgroup taxa. Number of specimens (N) and GenBank accession number are listed for each corresponding haplotype. Multiple specimens sharing the same haplotype are listed consecutively. All specimens collected from the USA except *P.nelsoni* Stark, 1989 (Canada). State or province is listed by standard postal abbreviation. Sequences obtained from GenBank are denoted with *. INHS = Illinois Natural History Survey Insect Collection record number.

Species	Sex	GenBank	N	INHS	Lat./ Long.	Stream	State/ Prov.	Collector(s)
*Beloneuriageorgiana* (Banks, 1914)	♂	MH778486	1	909265	34.69804N, -83.78149W	tributary of Dukes Creek	GA	E. J. South
*Perlestaadena* Stark, 1989	♂	MH778426	1	793345	36.39021N, -86.25096W	Rocky Creek	TN	S. A. Grubbs
*Perlestaarmitagei* Grubbs & DeWalt, 2018	♂	MH778427	1	457510	39.0342N, -86.16788W	Little Salt Creek	IN	R. E. DeWalt
*Perlestabjostadi* Kondratieff & Lenat, 2006	♂	MH778428	1	793346	36.84673N, -77.56095W	Nottoway River	VA	B. C. Kondratieff
*Perlestabrowni* Stark, 1989	♀	MH778429	1	658464	38.45202N, -92.48643W	South Moreau Creek	MO	E. J. South
*Perlestacinctipes* (Banks, 1905)	♂	MH778430	1	658465	38.45202N, -92.48643W	South Moreau Creek	MO	E. J. South
	♂	MH778431	1	658466	38.45202N, -92.48643W	South Moreau Creek	MO	E. J. South
	♀	MH778432	1	658467	38.45202N, -92.48643W	South Moreau Creek	MO	E. J. South
	♂	MH778433	1	658468	38.45202N, -92.48643W	South Moreau Creek	MO	E. J. South
*Perlestadecipiens* (Walsh, 1862)	♂	MH778434	1	658778	41.3337N, -88.18761W	Kankakee River	IL	A. Yanahan
	♀	MH778435	1	658777	41.3337N, -88.18761W	Kankakee River	IL	A. Yanahan
*Perlestaephelida* Grubbs & DeWalt, 2012	♂	MH778436	1	658780	44.72652N, -86.14303W	Platte River	MI	R. E. DeWalt, S. K. Ferguson
	♀	MH778437	1	658781	44.72652N, -86.14303W	Platte River	MI	R. E. DeWalt, S. K. Ferguson
	♂	MH778438	1	658469	37.49727N, -92.63033W	Osage Fork of Gasconade River	MO	E. J. South
	♂	MH778439	1	658470	37.49727N, -92.63033W	Osage Fork of Gasconade River	MO	E. J. South
	♀	MH778440	1	658477	37.49727N, -92.63033W	Osage Fork of Gasconade River	MO	E. J. South
*Perlestafrisoni* Banks, 1948	♂	*HQ568861	2	NA	35.62276N, -83.44288W	West Prong Little Pigeon River	TN	R. E. DeWalt
	♂	*JF884174	2	NA	35.4968N, -83.8337W	Twentymile Creek	NC	R. E. DeWalt
*Perlestagolconda* DeWalt & Stark, 1998	♂	MH778441	1	550392	41.67397N, -91.56452W	Iowa River	IA	M. Kippenhon
*Perlestalagoi* Stark, 1989	♂	MH778442	9	658456	38.45202N, -92.48643W	South Moreau Creek	MO	E. J. South
*Perlestalagoi* Stark, 1989	♂	MH778443	9	658457	38.45202N, -92.48643W	South Moreau Creek	MO	E. J. South
	♂	MH778444	9	658460	38.45202N, -92.48643W	South Moreau Creek	MO	E. J. South
	♂	MH778445	9	658461	38.45202N, -92.48643W	South Moreau Creek	MO	E. J. South
	♂	MH778446	9	658462	38.45202N, -92.48643W	South Moreau Creek	MO	E. J. South
	♀	MH778447	9	658471	38.45202N, -92.48643W	South Moreau Creek	MO	E. J. South
	♀	MH778448	9	658472	38.45202N, -92.48643W	South Moreau Creek	MO	E. J. South
	♀	MH778449	9	658473	38.45202N, -92.48643W	South Moreau Creek	MO	E. J. South
	♂	MH778450	9	658474	38.45202N, -92.48643W	South Moreau Creek	MO	E. J. South
	♂	MH778451	1	658459	38.45202N, -92.48643W	South Moreau Creek	MO	E. J. South
	♂	MH778452	1	658475	38.45202N, -92.48643W	South Moreau Creek	MO	E. J. South
	♂	MH778453	1	658476	38.45202N, -92.48643W	South Moreau Creek	MO	E. J. South
	♂	MH778454	1	658776	41.66065N, -81.11747W	Bates Creek	OH	E. J. South, R. E. DeWalt
*Perlestamihucorum* Kondratieff & Myers, 2011	♂	MH778455	1	793347	42.4401N, -73.8137W	Hannacroix Creek	NY	L. Myers, J. Myers
*Perlestanelsoni* Stark, 1989	♂	*KR144298	1	NA	45.976N, -66.719W	St. John River	NB	K. Heard et al.
*Perlestaouabache* Grubbs & DeWalt, 2011	♂	MH778456	1	516699	42.45994N, -89.23985W	Sugar River	IL	R. E. DeWalt et al.
*Perlestaroblei* Kondratieff & Kirchner, 2003	♂	MH778457	1	793348	36.4684N, -77.1443W	Kirbys Creek	NC	B. C. Kondratieff et al.
*Perlestasublobata* sp. nov.	♂	MH778458	8	793207	36.12052N, -93.69319W	War Eagle Creek	AR	E. J. South
	♂	MH778459	8	793212	34.03869N, -93.41752W	Antoine River	AR	E. J. South
	♂	MH778460	8	793218	34.17985N, -93.07021W	Caddo River	AR	E. J. South
	♂	MH778461	8	793230	36.04161N, -93.70482W	War Eagle Creek	AR	E. J. South
	♂	MH778462	8	793266	33.95608N, -93.44362W	Little Missouri River	AR	E. J. South
	♂	MH778463	8	793271	33.95608N, -93.44362W	Little Missouri River	AR	E. J. South
	♂	MH778464	8	793273	33.95608N, -93.44362W	Little Missouri River	AR	E. J. South
*Perlestasublobata* sp. nov.	♂	MH778465	8	793288	33.95608N, -93.44362W	Little Missouri River	AR	E. J. South
	♂	MH778466	1	793208	36.12052N, -93.69319W	War Eagle Creek	AR	E. J. South
	♂	MH778467	3	793209	35.66925N, -93.83033W	Mulberry River	AR	E. J. South
	♂	MH778468	3	793233	33.97121N, -94.22292W	Cossatot River	AR	E. J. South
	♀	MH778469	3	793324	33.95608N, -93.44362W	Little Missouri River	AR	E. J. South
	♂	MH778470	2	793211	35.66925N, -93.83033W	Mulberry River	AR	E. J. South
	♂	MH778471	2	793224	33.95608N, -93.44362W	Little Missouri River	AR	E. J. South
	♂	MH778472	1	793214	34.17985N, -93.07021W	Caddo River	AR	E. J. South
	♂	MH778473	1	793234	33.97121N, -94.22292W	Cossatot River	AR	E. J. South
	♂	MH778474	1	793299	33.95608N, -93.44362W	Little Missouri River	AR	E. J. South
	♂	MH778475	1	793312	33.95608N, -93.44362W	Little Missouri River	AR	E. J. South
	♀	MH778476	1	793328	33.95608N, -93.44362W	Little Missouri River	AR	E. J. South
	♀	MH778477	1	793331	33.95608N, -93.44362W	Little Missouri River	AR	E. J. South
*Perlestateaysia* Kirchner & Kondratieff, 1997	♂	MH778478	1	515560	39.1355N, -86.1601W	tributary of Middle Fork Salt Creek	IN	R. E. DeWalt
	♀	MH778479	1	457522	39.1355N, -86.1601W	tributary of Middle Fork Salt Creek	IN	R. E. DeWalt
*Perlesta* WI-1 (undescribed)	♂	MH778480	1	516410	46.07721N, -92.24608W	St. Croix River	WI	R. E. DeWalt et al.
	♀	MH778481	1	552631	46.07721N, -92.24608W	St. Croix River	WI	R. E. DeWalt, S. K. Ferguson
	♂	MH778482	1	576963	45.57953N, -87.78796W	Menominee River	WI	R. E. DeWalt et al.
	♂	MH778483	1	658779	45.77348N, -92.78164W	St. Croix River	MN	R. E. DeWalt
	♀	MH778484	1	583370	45.82306N, -92.77001W	Snake River	MN	R. E. DeWalt
*Perlestaxube* Stark & Rhodes, 1997	♂	MH778485	1	790543	39.40911N, -88.89952W	Mud Creek	IL	E. J. South, R. E. DeWalt
*Perlinelladrymo* (Newman, 1839)	♂	MH778487	1	514716	40.2942N, -87.2546W	Wabash River	IN	R. E. DeWalt, M. Pessino
*Perlinellaephyre* (Newman, 1839)	♂	MH778488	1	548835	42.32815N, -83.8595W	Huron River	MI	R. E. DeWalt et al.

We generated a maximum likelihood tree using MEGA 7.0 (Kumar et al. 2016) and calculated pairwise genetic distances for both sequences generated for this study, as well as additional sequences accessioned from GenBank, using the Kimura 2-parameter model (K2P) (Kimura 1980), the de facto standard for measuring mitochondrial pairwise distances (Collins et al. 2012). A Bayesian analysis was performed for all haplotypes using MrBayes 3.2.6 (Huelsenbeck and Ronquist 2001) with a burn-in length of 500,000, subsampling frequency of 500, and a chain length of 5,100,000.

## Results

### Perlesta sublobata

Taxon classificationAnimaliaPlecopteraPerlidae

South & DeWalt sp. nov.

http://zoobank.org/1FB6141B-3C6E-4B64-983C-406649DE6830

http://lsid.speciesfile.org/urn:lsid:Plecoptera.speciesfile.org:TaxonName:505368

**Figure 2.**
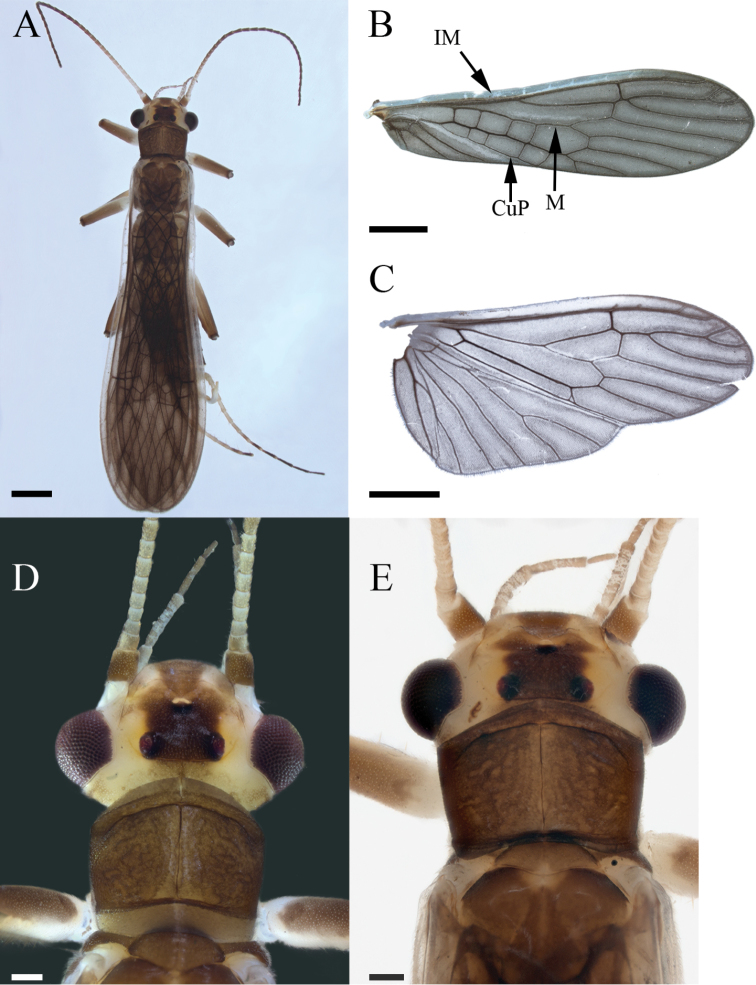
*Perlestasublobata* sp. nov., paratype males from Little Missouri River, Arkansas **A** habitus, dorsal view (INHS Insect Collection 793329) **B** dorsal view of right forewing showing intercostal margin (IM), posterior cubital vein (CuP), and median vein (M) **C** right hind wing, dorsal view (INHS Insect Collection 793270) **D** head and pronotum (INHS Insect Collection 793226) **E** head and pronotum (INHS Insect Collection 793329). Scale bars: 1 mm (**A**); 1.2 mm (**B, C**); 200 µm (**D, E**)

**Figure 3.**
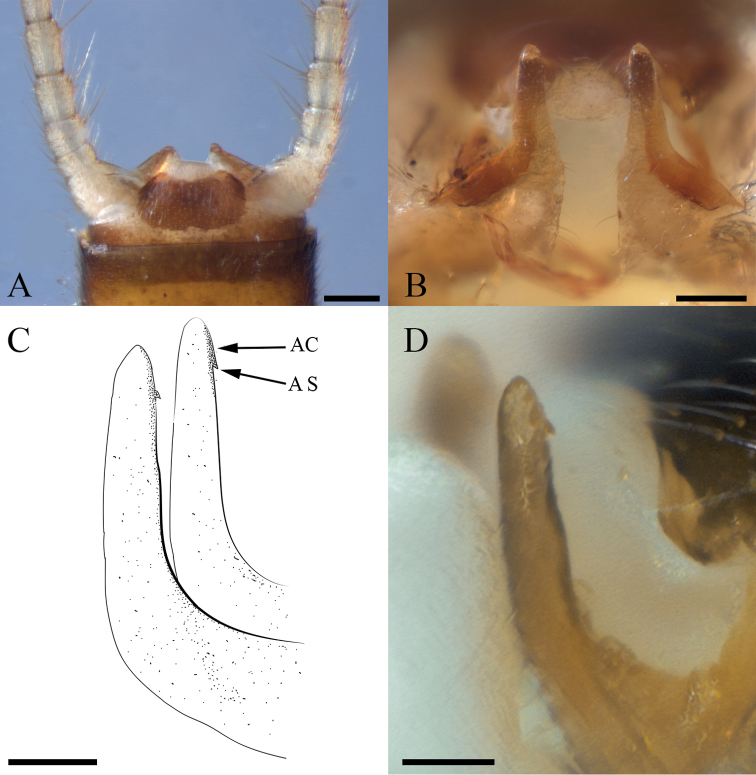
*Perlestasublobata* sp. nov., terminalia of paratype males from Little Missouri River, Arkansas **A** tenth tergite and paraprocts, dorsal view (INHS Insect Collection 793335) **B** paraprocts, caudal view **C** paraprocts, oblique lateral view showing anteromedially directed carina (AC) and spine (AS) **D** right paraproct of extruded male, oblique lateral view (INHS Insect Collection 793226). Scale bars: 200 µm (**A**); 100 µm (**B**), 50 µm (**C, D**).

**Figure 4.**
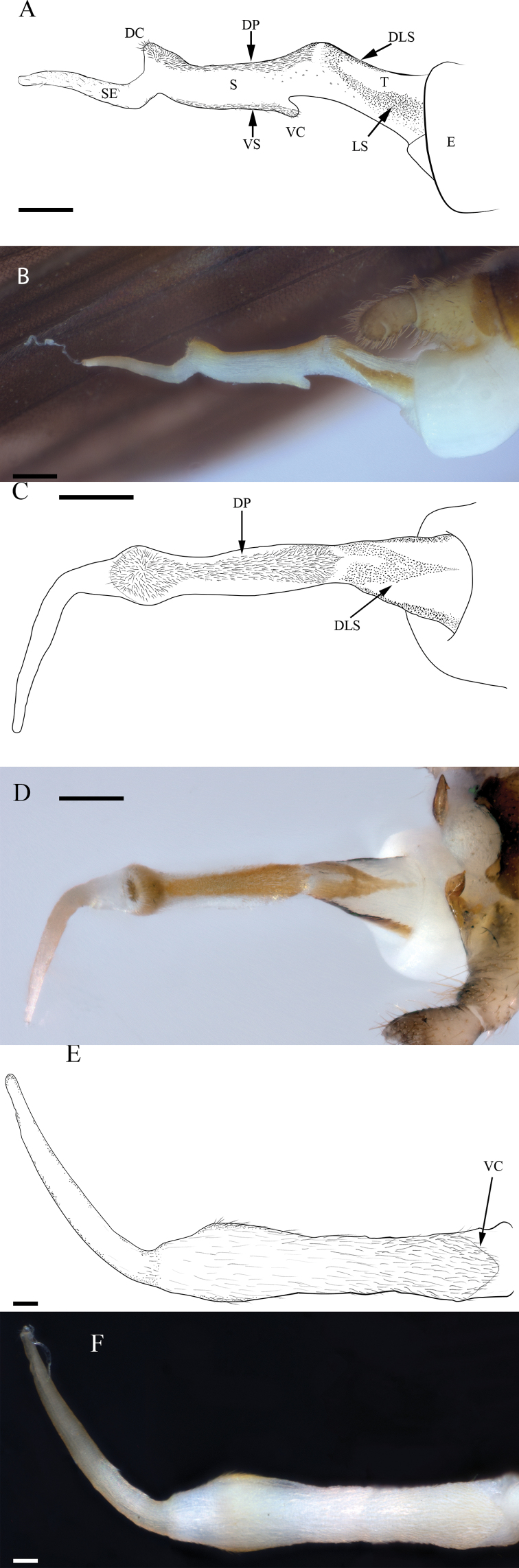
*Perlestasublobata* sp. nov., aedeagus of paratype male from Little Missouri River, Arkansas (INHS Insect Collection 793295) **A** lateral view showing dorsal caecum (DC), ventral caecum (VC), envelope (E), tube (T), sac (S), sac extension (SE), dorsal patch (DP), ventral seta-like spines (VS), lateral sclerite (LS), and dorsal extension of the lateral sclerites (DLS) **B** lateral view showing partially extruded dorsal caecum **C** dorsal view showing dorsal patch (DP) and dorsal extension of the lateral sclerites (DLS) **D** dorsal view showing partially extruded dorsal caecum **E** ventral view showing fine seta-like spines covering sac venter and ventral caecum (VC) **F** ventral view. Scale bars: 200 µm (**A, B, C, D**); 50 µm (**E**); 500 µm (**C, F**); 50 µm (**F**).

**Figure 5.**
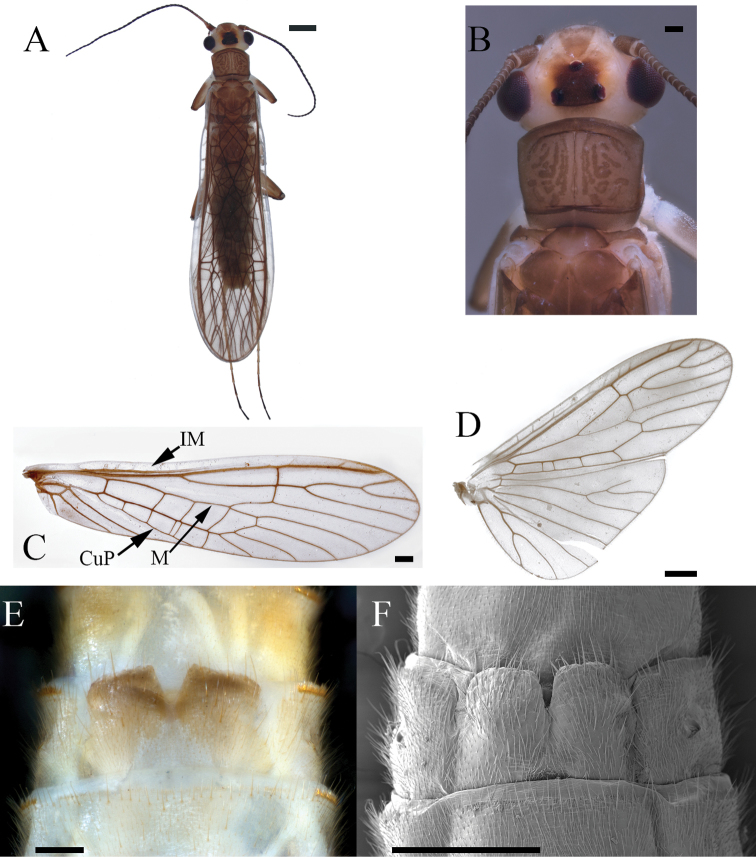
*Perlestasublobata* sp. nov., paratype females from Little Missouri River, Arkansas **A** habitus, dorsal view (scale bar) **B** head and pronotum (INHS Insect Collection 793329) (scale bar µm) **C** dorsal view of right forewing showing intercostal margin (IM), posterior cubital vein (CuP), and median vein (M) **D** right hind wing, dorsal view (INHS Insect Collection 793332) **E** subgenital plate (INHS Insect Collection 793329) **F** subgenital plate, SEM (INHS Insect Collection 793328). Scale bars: 1 mm (**A**); 200 µm (**B, E**); 500 µm (**C, F**); 750 µm (**D**).

**Figure 6.**
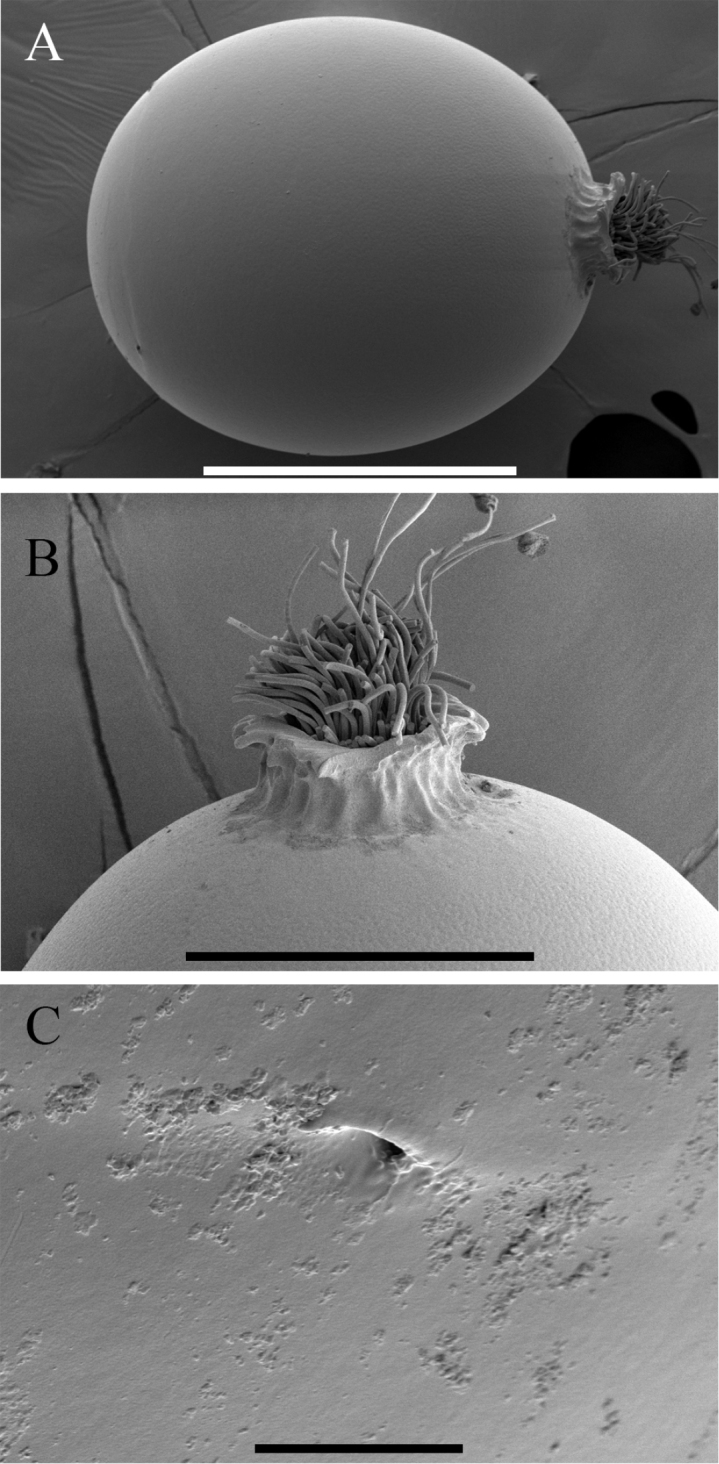
*Perlestasublobata* sp. nov., egg of paratype female from Little Missouri River, Arkansas (INHS Insect Collection 793316) **A** entire egg **B** posterior pole and collar **C** micropyle. Scale bars: 200 µm (**A**); 100 µm (**B**); 10 µm (**C**).

Figs 1 , 2 , 3 , 4 , 5 , 6

#### Diagnosis.

Males are distinguished by a combination of a prominent ventral caecum with a broad ventral setal patch and a distinct dorsal extension of the lateral sclerites of the aedeagus. Females possess a subgenital plate with a deep V-shaped notch and truncate lobes. Eggs have a smooth chorion and a well-developed, distally flanged collar.

**Male**. Habitus moderately dark (Fig. 2A). ***Wings***: Membrane brown with dark brown venation and pale intercostal margin (Fig. 2B, C). ***Forewing***: Length 8–9 mm (mean = 8.3 ± 0.3 SD, n = 95); membrane with two lightly pigmented longitudinal bands: one posterior to the posterior cubital vein and a second anterior to the median vein (Fig. 2B). ***Head***: Pale with dark brown quadrangular patch covering interocellar region; brown subtriangular patches anterolateral and anteromedial to median ocellus (Fig. 2D, E); diffuse brown pigmentation posterior to ecdysial suture (Fig. 2D); ecdysial suture extends slightly to moderately beyond ocelli as a distinct dark line; antenna darkly pigmented on ca. distal 2/3 of flagellum and dorsomedian region of scape; proximal antennal segments pale with tan dorsal pigmentation. ***Thorax***: Pronotum brown with vermiculated rugosities and faint, pale median stripe (Fig. 2D, E); mesothoracic and metathoracic nota brown; mesothoracic and metathoracic basisterna pale; femur and tibia pale, brown dorsally. ***Abdomen***: Sterna pale; terga pale medially and light brown laterally, or uniformly brown. ***Terminalia***: Tergum 10 with dark subquadrate pigment patch (Fig. 3A) and 10–20 small, sensilla basiconica (visible at 80× magnification); cercus long (holotype = 15 segments), pale proximally and dark brown distally; paraprocts broad basally and narrowed distally in caudal aspect (Fig. 3B); anteapical paraproct spine and carina directed anteromedially—best visible in oblique lateral view of unextruded individuals (Fig. 3C, D). ***Aedeagus***: Dorsal caecum moderately produced, ca. as long as wide and broad apically (Fig. 4A, B); dorsal patch broad over sac, moderately expanded proximally, constricted subapically, and broadly expanded over caecum; prominent lateral sclerites merge dorsally to form a distinct V-shaped pattern extending more than 1/2 tube length (Fig. 4C, D); prominent ventral caecum, narrowed apically, with a broad patch of fine seta-like spines covering venter and apex, length ca. 2/5 sac width; sac with fine seta-like spines covering venter (Fig. 4E, F).

**Female**. Female habitus similar to male, but of larger size and wings of lighter pigmentation (Fig. 5A). Pronotum tan with brown vermiculated rugosities and pale median stripe (Fig. 5B). Wings with subhyaline membrane, tan venation, and pale intercostal margin (Fig. 5C, D). Forewing length 9–11 mm (mean = 9.8 ± 0.6 SD, n = 40); often with two unpigmented longitudinal bands: one posterior to the posterior cubital vein and a second anterior to the median vein (Fig. 5C). Subgenital plate lobes truncate medially and truncate to slightly rounded laterally, slightly to moderately pigmented, covered with long bristle-like hairs, and separated by a deep V-shaped notch (Fig. 5E, F).

**Egg**. Length ca. 360 µm, width ca. 280 µm. Chorion smooth with fine pitting (Fig. 6A). Collar well developed, ribbed, and flanged distally (Fig. 6B). Micropylar orifices distinct near anterior pole (opposite collar) (Fig. 6C).

#### Molecular analyses.

**Figure 7.**
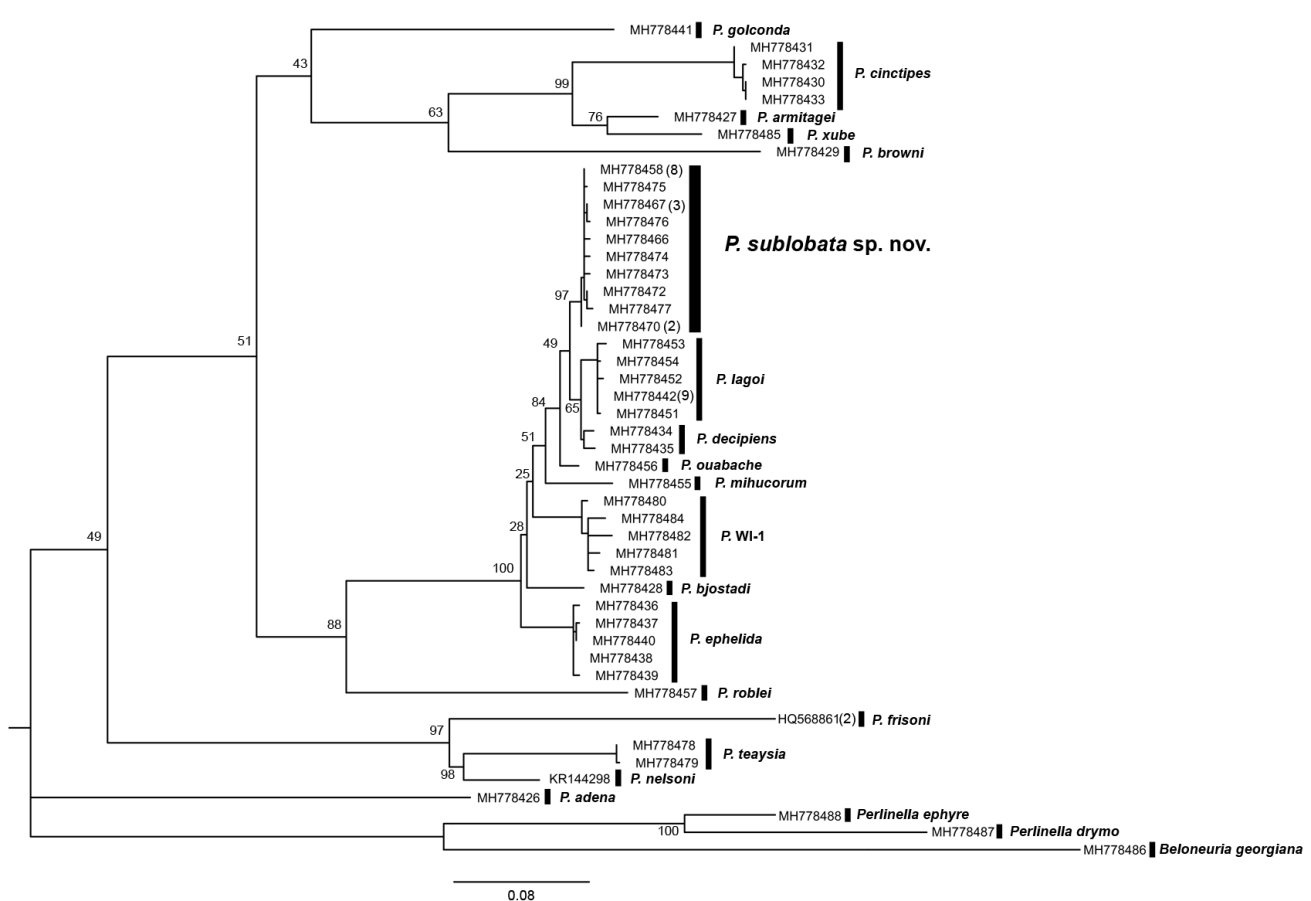
Maximum Likelihood phylogenetic reconstruction of 44 unique *Perlesta* CO1 haplotypes using the GTR+I+G nucleotide substitution model. Haplotypes represented by more than one specimen are indicated in parentheses beside corresponding GenBank accession numbers. Outgroup taxa: *Beloneuriageorgiana*, *Perlinelladrymo*, and *Perlinellaephyre*. Bootstrap scores from 1,000 replicates are displayed at nodes. Scale bar represents the estimated number of nucleotide substitutions per site.

**Figure 8.**
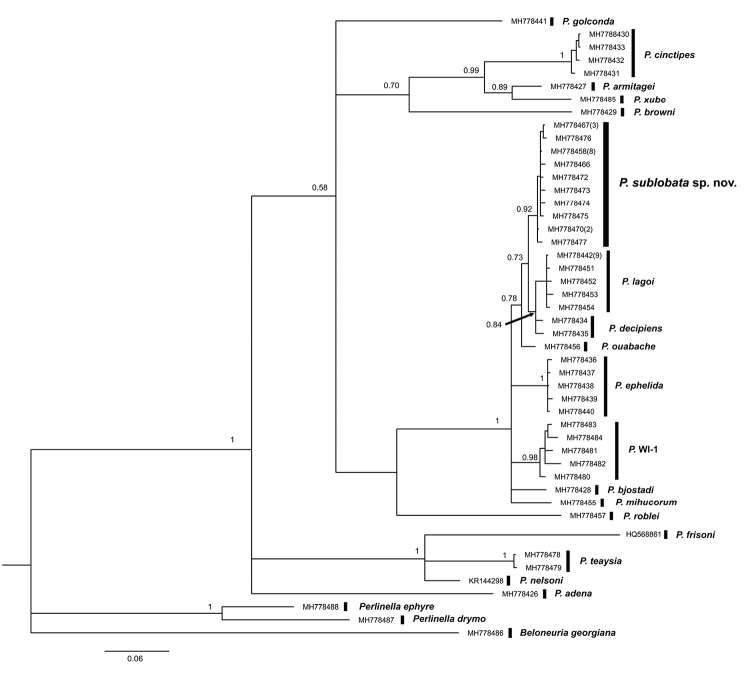
Bayesian phylogenetic reconstruction of 44 unique *Perlesta* CO1 haplotypes using the GTR+I+G nucleotide substitution model. Haplotypes represented by more than one specimen are indicated in parentheses beside corresponding GenBank accession numbers. Outgroup taxa: *Beloneuriageorgiana*, *Perlinelladrymo*, and *Perlinellaephyre*. Posterior probabilities are indicated at nodes. Scale bar represents the estimated number of nucleotide substitutions per site.

*Perlestasublobata* formed a monophyletic group with strong support (ML bootstrap support = 97%, Bayesian posterior probability = 92%). The nearest neighbor species to *P.sublobata* was *P.decipiens* (Walsh, 1862) at 1.8% sequence divergence. Maximum intraspecific COI genetic distances were less than minimum interspecific distances within all tested *Perlesta* (Table 2). All intraspecific distances were less than the arbitrary threshold of 3.5%, suggesting that the new species was monophyletic without other cryptic species present within the new taxon (Hebert et al. 2003, Zhou et al. 2010). All haplotypes (total = 47) were confined to their respective genera and presumptive species in the ML and Bayesian analyses (Figs 7, 8, respectively). The three tested species within the *P.frisoni* group, consisting of five Nearctic species that lack an aedeagal dorsal caecum, formed a monophyletic grouping. Four of the five “dark” species studied in Grubbs and DeWalt (2018) also formed a monophyletic grouping. The placement of *P.adena* Stark, 1989 outside this group may be spurious, indicating additional genes or populations are needed for further refinement. The relatively distant placement of *P.golconda* DeWalt & Stark, 1998 from *P.sublobata* is congruent with the species’ distinctly different morphologies, apart from the male genitalic similarities.

**Table 2.** Intra and interspecific distance. Maximum intraspecific and minimum interspecific (nearest neighbor) Kimura 2-parameter values for COI within *Perlesta*. Key: N = number of specimens, *P.* WI-1 is an undescribed species from Wisconsin.

Species	N	Maximum intraspecific distance (%)	Nearest neighbor	Nearest neighbor distance (%)
* P. adena *	1	0	* P. nelsoni *	19.3
* P. armitagei *	1	0	* P. xube *	7.2
* P. bjostadi *	1	0	* P. decipiens *	4.5
* P. browni *	1	0	* P. armitagei *	15.1
* P. cinctipes *	4	0.7	* P. armitagei *	10.4
* P. decipiens *	2	1.2	* P. lagoi *	1.5
* P. ephelida *	5	0.7	* P. ouabache *	4.8
* P. frisoni *	2	0	* P. nelsoni *	14.9
* P. golconda *	1	0	*P.* WI-1	17.8
* P. lagoi *	13	0.8	* P. decipiens *	1.5
* P. mihucorum *	1	0	* P. sublobata *	4.8
* P. nelsoni *	1	0	* P. teaysia *	9.4
* P. ouabache *	1	0	* P. sublobata *	2
* P. roblei *	1	0	* P. ephelida *	15.6
* P. sublobata *	20	0.8	* P. decipiens *	1.8
* P. teaysia *	2	0.2	* P. frisoni *	15.9
*P.* WI-1	5	2.4	* P. ouabache *	3.9
* P. xube *	1	0	* P. armitagei *	7.2

#### Remarks.

**Figure 9.**
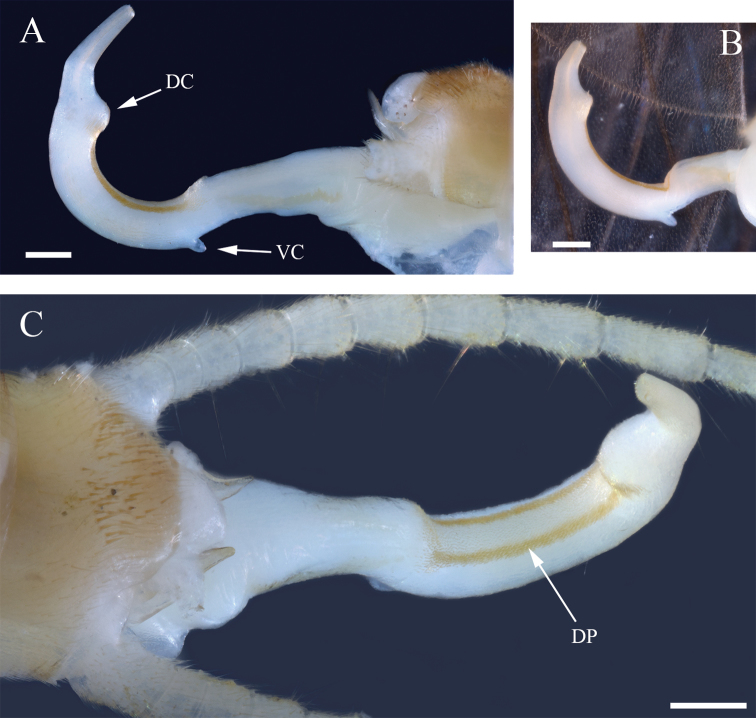
*Perlestagolconda*, aedeagi of males from Missouri River, Nebraska **A** lateral view showing dorsal caecum (DC) and ventral caecum (VC) (INHS Insect Collection 660209) **B** lateral view (INHS Insect Collection 660210) **C** dorsal view showing dorsal patch (DP) (INHS Insect Collection 660209). Scale bars: 200 µm.

The shape and armature of the aedeagus are the most distinct morphological features of *P.sublobata*. Stark (1989) illustrated a lateral view of an undetermined species from Arkansas (*P.sublobata*), demonstrating spinule patterns and shape of the aedeagal telescoping sections: envelope, tube, and sac. He noted that lateral sclerites of the tube joined dorsally. This dorsal extension of the lateral sclerites was not illustrated or specified in the literature for any other *Perlesta*. Furthermore, a ventral caecum is present in *P.sublobata* and only one other described congener, *P.golconda*. However, the ventral caecum of *P.golconda* is less prominent and without a distinct ventral patch of fine seta-like spines. Additionally, the dorsal caecum of *P.sublobata* is moderately developed, compared to the poorly developed dorsal caecum of *P.golconda* (Fig. 9).

**Figure 10.**
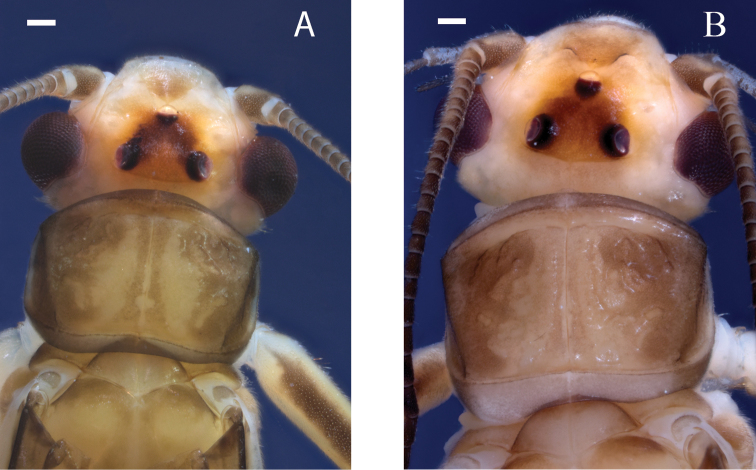
*Perlestagolconda*, head and pronotum, Missouri River, Nebraska **A** male (INHS Insect Collection 660210) **B** female (INHS Insect Collection 658790). Scale bars: 200 µm.

**Figure 11.**
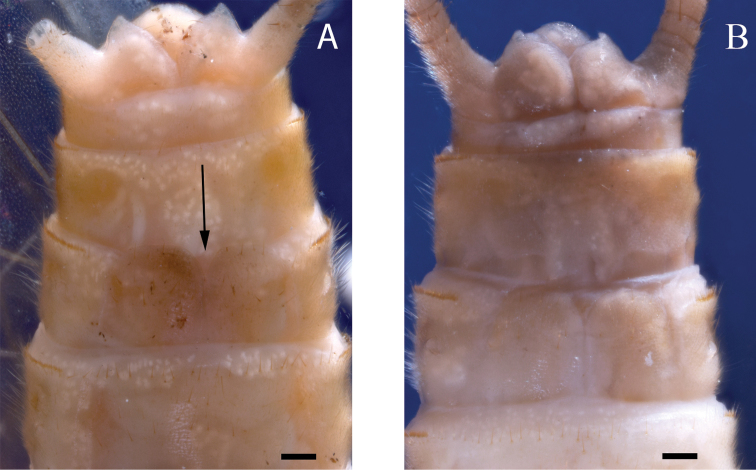
*Perlestagolconda*, subgenital plates of females from Missouri River, Nebraska **A** notch indicated by arrow (INHS Insect Collection 658788) **B** (INHS Insect Collection 658789). Scale bars: 200 µm.

The known distribution of *P.golconda*, originally limited to Illinois (DeWalt and Stark 1998), has expanded to include Iowa, Indiana, Michigan, and Nebraska (DeWalt et al. 2019), as well as Missouri (Stark 2004) and Louisiana (INHS Insect Collection 564765). Arkansas is bordered by Missouri to the north and Louisiana to the south. A sympatric distribution with *P.sublobata* is expected due to this geographic adjacency and overlap of the Interior Highlands’ habitat. Consequently, re-examination of some museum specimens may be required. The male and female habitus easily distinguish *P.golconda* from *P.sublobata*. The ocelli of *P.golconda* are usually connected by a moderately dark V-shaped pattern on a pale background (Fig. 10A, B), whereas *P.sublobata* has a dark subquadrate interocellar region. The pronotum of *P.golconda* is primarily pale with light tan rugosities on the lateral margins, whereas *P.sublobata* has a dark pronotum with a pale narrow median stripe. Additionally, *P.golconda* females are distinguished by a very short egg collar (Grubbs and DeWalt 2008, their fig. 17) and rounded subgenital plate lobes (Fig. 11A, B).

**Figure 12.**
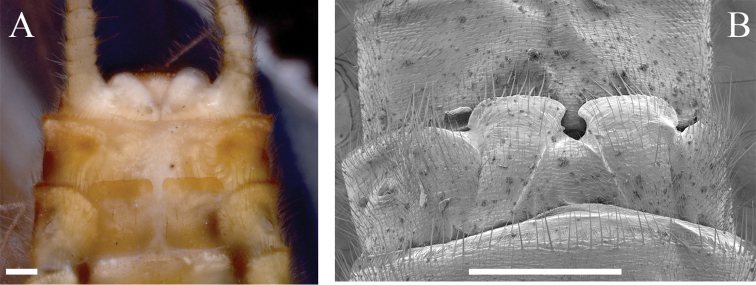
*Perlestadecipiens*, female subgenital plates **A** Kankakee River, Illinois (INHS Insect Collection 577949) **B** Caddo River, Arkansas, SEM (INHS Insect Collection 793908). Scale bars: 200 µm (**A**); 500 µm (**B**).

**Figure 13.**
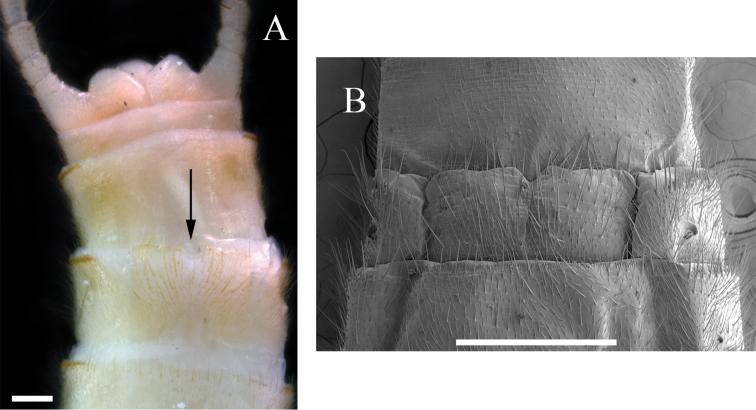
*Perlestaephelida*, female subgenital plates **A** Platte River, Michigan, notch indicated by arrow (INHS Insect Collection 658781) **B** Sugar Creek, Indiana, SEM (INHS Insect Collection 658791). Scale bars: 200 µm (**A**); 500 µm (**B**).

The female habitus of *P.sublobata* resembles two Interior Highlands congeners, *P.decipiens* and *P.ephelida* Grubbs and DeWalt, 2012. However, *P.sublobata* differs from *P.decipiens* and *P.ephelida* by subgenital plate morphology. *Perlestadecipiens* has a deep U-shaped notch bordered by truncate lobes, typically with darker pigmentation on the posterior margins (Fig. 12). *Perlestaephelida* has a shallow V-shaped notch enclosed by truncate lobes, usually pale to lightly pigmented with posteromedially upturned margins (Fig. 13; Grubbs and DeWalt 2012, their fig. 7). These characters are contrasted to the deep V-shaped notch and moderately pigmented, truncate lobes of *P.sublobata*. Furthermore, *P.sublobata* has a shorter forewing length than *P.decipiens* (*P.sublobata* = 9–11 mm; *P.decipiens* = 12–13 mm, Stark 2004). Egg chorion and collar are similar to *P.decipiens* (Stark 2004, his figs 7.397–7.399) and *P.ephelida* (Grubbs and DeWalt 2012, their figs 14–21).

#### Habitat.

**Figure 14.**
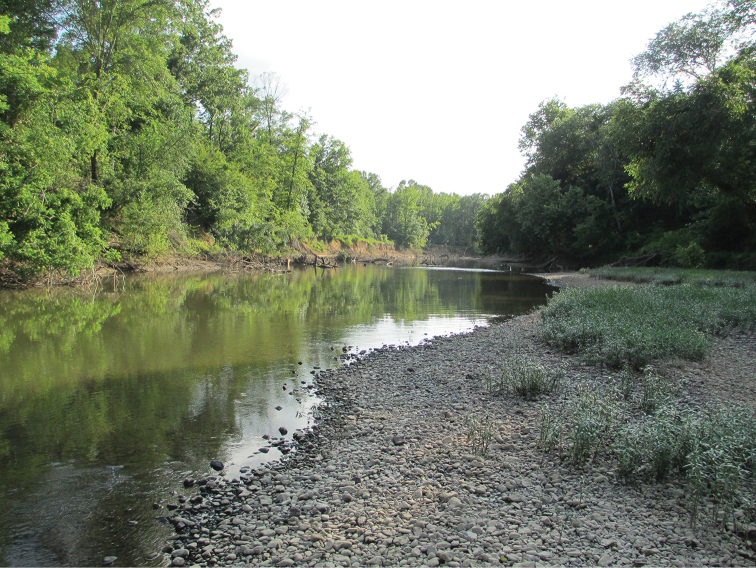
Little Missouri River, Pike County, Arkansas, USA. Type locality for *Perlestasublobata* sp. nov.

With the exception of one locality (OK, Washington Co., Caney River), all collection sites for *P.sublobata* are within or closely adjacent to the Interior Highlands, a region containing four contiguous U. S. Environmental Protection Agency (EPA) Level III Ecoregions: Ozark Highlands, Boston Mountains, Arkansas Valley, and Ouachita Mountains. Collection sites for *P.sublobata* within the Interior Highlands are partially canopied, hardwood forested, wadeable, low gradient streams (ca. 15–20 m wide) with substrata composed mostly of sand, gravel, and cobble. The type locality is a low gradient run (ca. 25 m wide) of the Little Missouri River (Fig. 14), located 45 km downstream of Lake Greeson and 65 km upstream from its confluence with the Ouachita River in the extreme north EPA Level III Ecoregion 35 (South Central Plains). The substrate is primarily gravel and sand, with some large woody debris. Other stonefly species collected with the new species at the type locality included *Acroneuriafrisoni* Stark & Brown, 1991, Acroneurianr.ozarkensis Poulton & Stewart, 1991, *Agnetinaflavescens* (Walsh, 1862), *Neoperlafalayah* Stark & Lentz, 1988, *N.robisoni* Poulton & Stewart, 1986, *P.decipiens*, and *Perlinellaephyre* (Newman, 1839).

#### Etymology.

The specific epithet is derived from *sub*, Latin for under, and *lobata*, the feminine adjectival form of *lobus*, Latin for a rounded projection or protuberance (Brown 1956). The name references the ventral caecum of the aedeagus, a character shared by only one other described congener, *P.golconda*, though it is most prominent in *P.sublobata*.

### *Material examined: Perlestasublobata*

**Holotype**: ♂, in 95% ethanol, **USA: Arkansas**: Pike Co., Little Missouri River, 10.0 km SSE Delight at AR-19, 33.95608, -93.44362, 15.vi.2016, E. J. South (INHS Insect Collection 793224).

**Paratypes. USA: Arkansas**: Clark Co., Caddo River, 6.7 km NNW Arkadelphia at Super 8 Motel at US-67, 34.17985, -93.07021, 14.vi.2016, E. J. South, 6♂ (INHS Insect Collection 793213–793218); Franklin Co., Mulberry River, 2.3 km SSW Cass at AR-23, 35.66984, -93.82962, 13.vi.2016, E. J. South, 3♂ (INHS Insect Collection 793209–793211); Madison Co., War Eagle Creek, 5.8 km NE Huntsville at AR-412, 36.12076, -93.69354, 13.vi.2016, E. J. South, 3♂ (INHS Insect Collection 793206–793208), same but 16.vi.2016, E. J. South, ♂ (INHS Insect Collection 793228), same but 17.vi.2016, E. J. South, 4♂ (INHS Insect Collection 793229–792232); Pike Co., Antoine River, Antoine at AR-26, 34.03899, -93.41803, 14.vi.2016, E. J. South, ♂ (INHS Insect Collection 793212). Same data as holotype, E. J. South 10♂ (INHS Insect Collection 793219–793227), same but 18.vi.2016, E. J. South, 71♂, 20♀ (INHS Insect Collection 793261–793343); Sevier Co., Cossatot River, 5.1 km W Lockesburg at AR-24, 33.97145, -94.22274, 18.vi.2016, E. J. South, 9♂, 19♀ (INHS Insect Collection 793233–793260).

#### Other material examined.

**USA: Arkansas**: Franklin Co., Mulberry River, Hwy 23 at Turner’s Bend, 35.66984, -93.82962, 5.vii.1986, B. C. Poulton, 4♂ (INHS Insect Collection 795241); Howard Co., Cossatot River, 12.9 km W Umpire at Hwy 4, 34.29584, -94.17787, 26.vi.1981, H. W. Robison, 10♂ (INHS Insect Collection 794630), same but Saline River, 8 km S Umpire at Hwy 4, 34.21096, -94.05099, 9.vii.1982, H. W. Robison, D. Koym, 7♂, 5♀ (INHS Insect Collection 794640), same but 1.6 km W Athens at Hwy 84, 34.31498, -93.99048, 9.vii.1984, H. W. Robison, D. Koym, 3♂, 10♀ (INHS Insect Collection 794629, 794634); Johnson Co., Mulberry River, 4.8 km W Ozark at Wolf Pen, 35.67376, -93.63271, 16.vii.1983, H. W. Robison, D. Koym, 8♂ (INHS Insect Collection 794643); Madison Co., War Eagle Creek, 4.8 km NE Huntsville at Hwy 68, 36.12076, -93.69354, 27.v.1978, J. McGraw, 17♂ (INHS Insect Collection 794636); Nevada Co., Little Missouri River, 17.7 km N Prescott at AR-19, 33.95571, -93.44388, 3.vii.1982, D. Koym, 8♂, 11♀ (INHS Insect Collection 794641), Little Missouri River, Nubbin Hill Rd., 33.93804, -93.35393, 1.vi.1982, D. Koym, 8♂ (INHS Insect Collection 794637); Pike Co., Antoine River, Antoine at AR-26, 34.03899, -93.41803, 18.vi.1982, D. Koym, 10♂, 8♀ (INHS Insect Collection 794638); Saline Co., Middle Fork Saline River, 1.6 km NW Owensville, 34.63066, -92.82711, 10.vii.1981, H. W. Robison, S. Harris, 4♂ (INHS Insect Collection 794631); Scott Co., Shadley Creek, 0.4 km S Bates, 34.90626, -94.38661, 12.vi.1983, H. W. Robison, D. Koym, 10♂ (INHS Insect Collection 794642); Sevier Co., Cossatot River, AR-24, 33.97145, -94.22274, 26.vi.1982, H. W. Robison, 10♂ (INHS Insect Collection 794632); Van Buren Co., South Fork Little Red River, 4 km NE Scotland at AR-95, 35.54868, -92.58541, 22.vi.1985, H. W. Robison, 15♂ (INHS Insect Collection 793774); Washington Co., Cove Creek, 24.1 km S Prairie Grove, 35.79531, -94.36519, 6.vi.1962, O. Hite, M. Hite, 2♂, 2♀ (INHS Insect Collection 794639). **Oklahoma**: Atoka Co., motel, Atoka, 34.38538, -96.12788, 4.vi.1969, D. C. Arnold, 2♂ (OKSU Midwest Plecoptera 19534); Le Flore Co., Big Creek, Page, 34.71595, -94.55016, 23.vi.1937, Standish, Kaiser, 8♂ (OKSU Midwest Plecoptera 19529); McCurtain Co., Broken Bow, 34.02983, -94.73871, 29.vii.1937, Standish, Kaiser, 34♂, 30♀ (OKSU Midwest Plecoptera 19517, 19518), Sherwood, 34.33121, -94.77833, 27.vi.1937, Standish, Kaiser, 5♂, 24♀ (OKSU Midwest Plecoptera 19521, 19522), West Fork Glover River, Battiest, 34.39393, -94.94166, 14.vi.1972, D. C. Arnold, 2♂ (OKSU Midwest Plecoptera 19523), Mountain Fork, Beaver’s Bend State Park, 34.13960, -94.70704, 11.vi.1985, D. C. Arnold, 3♂ (OKSU Midwest Plecoptera 19526, 195277), same but 10.vi.1985, D. C. Arnold, ♂, 4♀ (OKSU Midwest Plecoptera 19528); Pontotoc Co., Ada, 34.77447, -96.67892, 16.vii.1937, Standish, Kaiser, 2♂, 2♀ (OKSU Midwest Plecoptera 19519, 19520); Washington Co., Caney River, Bartlesville, 36.75401, -95.97137, 31.v.1978, D. C. Arnold, ♂ (OKSU Midwest Plecoptera 19525).

### Material examined: *Perlestagolconda*

**USA: Illinois**: Carroll Co., Mississippi River, Savanna, 42.09622, -90.16227, 19.vi.1999, R. E. DeWalt, ♂ (INHS Insect Collection 566462). **Indiana**: Ohio Co., Arnold Creek, 6.9 km WSW Rising Sun at IN-262 and White Rd., 38.93676, -84.93167, 14.v.2018, E. A. Newman, ♀ (INHS Insect Collection 660320). **Iowa**: Cedar Co., Cedar River, Cedar Bluff at Hwy F28, 41.78790, -91.31340, 2.viii.2000, D. Heimdal, ♂, 2♀ (INHS Insect Collection 36061). **Louisiana**: East Baton Rouge Co., Mississippi River, Baton Rouge at Centroplex Pier N I-10Br., 30.44532, -91.19184, 12.vi.1992, R. E. DeWalt, ♂, ♀ (INHS Insect Collection 564765). **Minnesota**: Winona Co., Mississippi River, 3.6 km N La Crescent, rest stop at I-90, 43.85981, -91.30351, 18.vi.2012, R. E. DeWalt, ♀ (INHS Insect Collection 577372). **Nebraska**: Nemaha Co., Missouri River, Brownville, 200 m downstream US-136, 40.39335, -95.64948, 17.vi.2018, R. E. DeWalt, 6♂, ♀ (INHS Insect Collection 660253, 660254), same but 24.vi.2018, R. E. DeWalt, 8♂, 10♀ (INHS Insect Collection 660209–660220).

### Modified key to first couplet in Stark (2004) for identification of males of *Perlestasublobata* and *P.golconda*

**Table:** 

1	Fully everted aedeagus with dorsal caecum (fig. 7.273)	**1a**
–	Fully everted aedeagus without dorsal caecum (fig. 7.361)	**17**
1a	Aedeagus with ventral caecum	**1b**
–	Aedeagus without ventral caecum	**2**
1b	Ventral caecum prominent, length ca. 2/5 aedeagal sac width, fine ventral seta-like spines present; dorsal caecum moderately developed (Fig. 4A); distinct dorsal extension of the aedeagal lateral sclerites with proximal V-shaped pattern (Fig. 4C); ocellar area with dark subquadrate patch	*** P. sublobata ***
–	Ventral caecum less prominent, length ca. 1/3 aedeagal sac width, without distinct fine ventral seta-like spines; dorsal caecum poorly developed; dorsal aedeagal patch with lateral margins darker than mesal field, appearing as two tracks (fig.7.364); ocelli usually connected by V-shaped area with pale center	*** P. golconda ***

## Supplementary Material

XML Treatment for Perlesta sublobata
